# 囊腔型肺腺癌临床多特征分析及浸润性风险预测模型的构建

**DOI:** 10.3779/j.issn.1009-3419.2024.102.14

**Published:** 2024-04-20

**Authors:** Qiang WANG, Chenghao FU, Kun WANG, Qianrui REN, Aiping CHEN, Xinfeng XU, Liang CHEN, Quan ZHU

**Affiliations:** ^1^210029 南京，南京医科大学第一附属医院胸外科; ^1^Department of Thoracic Surgery, The First Affiliated Hospital of Nanjing Medical University, Nanjing 210029, China; ^2^225300 泰州，泰州市第四人民医院胸外科; ^2^Department of Thoracic Surgery, Taizhou Fourth People's Hospital, Taizhou 225300, China; ^3^210029 南京，南京医科大学第一附属医院放射科; ^3^Department of Radiology, The First Affiliated Hospital of Nanjing Medical University, Nanjing 210029, China

**Keywords:** 囊腔, 肺肿瘤, 浸润性, 预测模型, Cyst, Lung neoplasms, Invasiveness, Prediction model

## Abstract

**背景与目的:**

囊腔型肺癌作为一种特殊类型的肺癌逐步得到人们的关注，其最常见的病理类型为腺癌。囊腔型肺腺癌的浸润性对诊疗方案的选择和预后至关重要。本研究旨在分析囊腔型肺腺癌临床多特征，探讨其浸润性的独立危险因素并建立风险预测模型。

**方法:**

回顾性分析2021年1月至2022年7月于南京医科大学第一附属医院胸外科行手术治疗的129例囊腔型肺腺癌患者，根据病理结果分成浸润前组：非典型腺瘤样增生（atypical adenomatous hyperplasia, AAH）、原位腺癌（adenocarcinoma in situ, AIS）、微浸润型腺癌（minimally invasive adenocarcinoma, MIA）与浸润组：浸润性腺癌（invasive adenocarcinoma, IAC）。其中浸润前组47例，男性19例，女性28例，平均年龄（51.23±14.96）岁；浸润组82例，男性60例，女性22例，平均年龄（61.27±11.74）岁。收集两组病例多组临床特征，采用单因素分析、LASSO回归、多因素Logistic回归分析得出囊腔型肺腺癌浸润性的独立危险因素，建立浸润性风险预测模型。

**结果:**

单因素分析显示年龄、性别、吸烟史、肺气肿、神经元特异性烯醇化酶（neuron-specific enolase, NSE）、囊腔数、病灶直径、囊腔直径、结节直径、实性成分直径、囊壁结节、囊壁光滑程度、囊腔形状、分叶征、短毛刺征、胸膜牵拉、血管穿行与支气管穿行在囊腔型肺腺癌浸润前组与浸润组间存在统计学差异（P<0.05）。上述变量经LASSO回归降维处理，进一步筛选出的变量包括：年龄、性别、吸烟史、NSE、囊腔数、病灶直径、囊腔直径、囊壁结节、囊壁光滑程度与分叶征，并纳入多因素Logistic回归分析，发现囊壁结节（P=0.035）与分叶征（P=0.001）是囊腔型肺腺癌浸润性的独立危险因素（P<0.05）。建立预测模型如下：P=e^x/（1+e^x），x=-7.927+1.476*囊壁结节+2.407*分叶征，曲线下面积（area under the curve, AUC）为0.950。

**结论:**

囊壁结节及分叶征为囊腔型肺腺癌浸润性的独立危险因素，对囊腔型肺腺癌的浸润性预测具有一定的指导意义。

## Abstract (en)

**Background and objective:**

Cystic lung cancer, a special type of lung cancer, has been paid more and more attention. The most common pathological type of cystic lung cancer is adenocarcinoma. The invasiveness of cystic lung adenocarcinoma is vital for the selection of clinical treatment and prognosis. The aim of this study is to analyze the multiple clinical features of cystic lung adenocarcinoma, explore the independent risk factors of its invasiveness, and establish a risk prediction model.

**Methods:**

A total of 129 cases of cystic lung adenocarcinoma admitted to the Department of Thoracic Surgery of the First Affiliated Hospital of Nanjing Medical University from January 2021 to July 2022 were retrospectively analyzed and divided into pre-invasive group [atypical adenomatous hyperplasia (AAH), adenocarcinoma in situ (AIS) and minimally invasive adenocarcinoma (MIA)] and invasive group [invasive adenocarcinoma (IAC)] according to pathological findings. There were 47 cases in the pre-invasive group, including 19 males and 28 females, with an average age of (51.23±14.96) years. There were 82 cases in the invasive group, including 60 males and 22 females, with an average age of (61.27±11.74) years. Multiple clinical features of the two groups were collected, including baseline data, imaging data and tumor markers. Univariate analysis, LASSO regression and multivariate Logistic regression analysis were used to screen out the independent risk factors of the invasiveness of cystic lung adenocarcinoma, and the risk prediction model was established.

**Results:**

In univariate analysis, age, gender, smoking history, history of emphysema, neuron-specific enolase (NSE), number of cystic airspaces, lesion diameter, cystic cavity diameter, nodule diameter, solid components diameter, cyst wall nodule, smoothness of cyst wall, shape of cystic airspace, lobulation, short burr sign, pleural retraction, vascular penetration and bronchial penetration were statistically different between the pre-invasive group and invasive groups (P<0.05). The above variables were processed by LASSO regression dimensionality reduction and screened as follows: age, gender, smoking history, NSE, number of cystic airspaces, lesion diameter, cystic cavity diameter, cyst wall nodule, smoothness of cyst wall and lobulation. Then the above variables were included in multivariate Logistic regression analysis. Cyst wall nodule (P=0.035) and lobulation (P=0.001) were found to be independent risk factors for the invasiveness of cystic lung adenocarcinoma (P<0.05). The prediction model was established as follows: P=e^x/(1+e^x), x=-7.927+1.476* cyst wall nodule+2.407* lobulation, and area under the curve (AUC) was 0.950.

**Conclusion:**

Cyst wall nodule and lobulation are independent risk factors for the invasiveness of cystic lung adenocarcinoma, which have certain guiding significance for the prediction of the invasiveness of cystic lung adenocarcinoma.

随着影像技术的发展，计算机断层扫描（computed tomography, CT）广泛应用于肺部肿瘤的筛查，肺癌病灶检出率不断增高，囊腔型肺癌这种特殊类型的肺癌逐步得到关注。其影像学表现为在肺癌病灶的基础上存在囊腔样改变，且内部或周边存在实性或非实性结节样成分^[1]^。Womack等^[2]^于19世纪40年代首次报道了该类型肺癌，随即Anderson等^[3]^多位学者对其进一步深入研究。2022年美国放射学院修订了肺部影像和报告系统（Lung Imaging Reporting and Data System, Lung-RADS）分级^[4]^，将囊腔型病灶列为新的分类，纳入分级管理。囊腔型肺癌发病率仅占肺癌总比的3.7%-9.3%^[5,6]^，预后较同分期非囊腔型肺癌患者要差^[7]^，且22.7%的囊腔型肺癌被遗漏或延迟诊断^[8]^。因此，囊腔型肺癌需要临床医师予以高度重视。

囊腔型肺腺癌是最常见的囊腔型肺癌^[9]^，其包含非典型腺瘤样增生（atypical adenomatous hyperplasia, AAH）、原位腺癌（adenocarcinoma in situ, AIS）、微浸润型腺癌（minimally invasive adenocarcinoma, MIA）与浸润性腺癌（invasive adenocarcinoma, IAC）四种病理亚型，浸润性逐步增加。IAC预后较差，而其他亚型5年生存率几乎为100%^[10]^。因此，浸润性高低对于囊腔型肺腺癌的诊治具有直接意义。目前国内针对囊腔型肺腺癌的临床研究主要集中在良恶性简单对照与CT典型特征探究上，尚未见对囊腔型肺腺癌浸润性的术前预测与独立危险因素分析。本研究收集2189例肺部手术患者资料，从中筛选出129例囊腔型肺腺癌患者，探讨浸润性的独立危险因素，并建立浸润性风险预测模型，为囊腔型肺腺癌的早期筛查及诊断提供理论依据。

## 1 资料与方法

### 1.1 病例选择

囊腔型肺腺癌定义为术后病理明确为肺腺癌基础上，影像学表现存在囊腔样改变，且内部或周边存在实性或非实性结节成分。选择2021年1月至2022年7月于南京医科大学第一附属医院胸外科行肺部手术的患者共计2189例。纳入标准：（1）年龄≥18岁；（2）术后病理明确为原发性肺腺癌患者；（3）术前影像学表现为囊腔型病灶。排除标准：（1）手术目标病灶非囊腔型肺腺癌病灶；（2）多发病灶；（3）临床资料缺失；（4）术前接受新辅助治疗。经过筛查，共129例囊腔型肺腺癌患者纳入研究，见图1。

**图 1:**
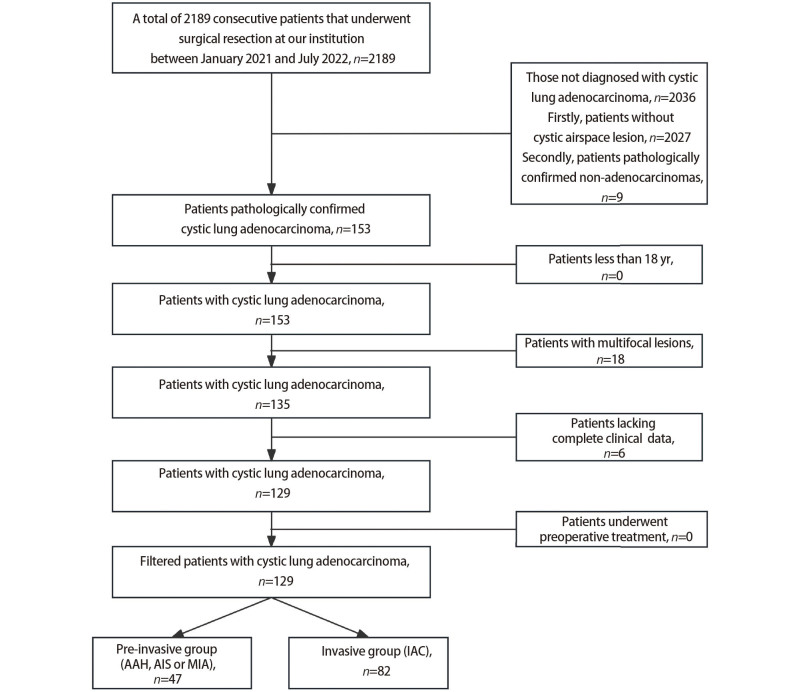
囊腔型肺腺癌患者纳入流程图

### 1.2 病理学评估与分组

术后病理由具有3年以上经验的病理医生根据2011年国际肺癌研究学会（International Association for the Study of Lung Cancer, IASLC）/美国胸科学会（American Thoracic Society, ATS）/欧洲呼吸协会（European Respiratory Society, ERS）分类系统^[10]^进行诊断和分类，并由另一位具有10年以上经验的病理医生进行审查。诊断的腺癌亚型分为AAH、AIS、MIA与IAC。依据病理结果分为浸润前组（AAH、AIS、MIA）与浸润组（IAC）。

### 1.3 CT图像的获取

所有CT扫描均使用西门子128层螺旋CT扫描（Siemens, Definition AS+; Malvern, Pa），层厚1.5 mm，层间距1.2 mm。参数设置：电压100或120 kVp，mAs设置应用CARE Dose 4D个体化选择，扫描矩阵512×512。窗宽和窗位：肺窗（窗宽：1200 HU；窗位：-600 HU），纵隔窗（窗宽：350 HU；窗位：50 HU）。

### 1.4 CT特征判读、指标测量与病灶分类

所有入组患者的术前胸部CT资料均传送至影像工作站进行处理分析，采用多截面重建（multiple planes reconstruction, MPR）等多种处理技术进行判读与测量。两名有10年以上经验的胸部放射科医生，独立解释CT图像；两人诊断意见不统一时，经协商解决意见分歧。我们集中对11项特征展开判读，包括：病灶位置（左上/左下/右上/右中/右下叶）、病灶分带（外/中/内）、囊腔数（单/多）、囊壁结节（有/无）、囊壁光滑程度（光滑/粗糙）、囊腔形状（规则/不规则）、分叶征（有/无）、短毛刺征（有/无）、胸膜牵拉（有/无）、血管穿行（有/无）、支气管穿行（有/无）；对5项指标展开测量，包括：深度比（所处深度带）、病灶直径、囊腔直径、囊壁结节直径与实性成分直径。深度比的测量参照了王俊教授团队的研究成果^[11]^，将测量值按1/3划分，分别代表结节所处外/中/内带。病灶直径测量为肺窗上病灶（包含囊腔部分）在MPR的最大轴向截面的最大直径。囊腔直径测量为囊腔在MPR的最大轴向截面的最大直径；如果病灶包含几个囊腔，则测量最大囊腔直径。对于囊壁结节直径与实性成分直径的测量，我们根据Mascalchi^[12]^、Fintelmann^[13]^等学者提出的形态学分类系统，将囊壁结节分为环壁型结节与团块型结节两类，提出以下测量方法：环壁型结节处理为从囊腔边界到结节边界的最大距离，垂直于囊腔边界切线，类似于环壁型结节的壁厚；团块型结节处理为团块型结节在MPR的最大轴向截面的最大直径。根据Ko等^[14]^研究，定义病灶实性成分CT界定值为-188 HU，实性成分直径的测量同样采用上述囊壁结节直径测量的方法进行，如果病灶包含几个实性成分，则测量最大实性成分直径。本研究囊腔型肺腺癌影像学测量方法如图2所示。

**图 2:**
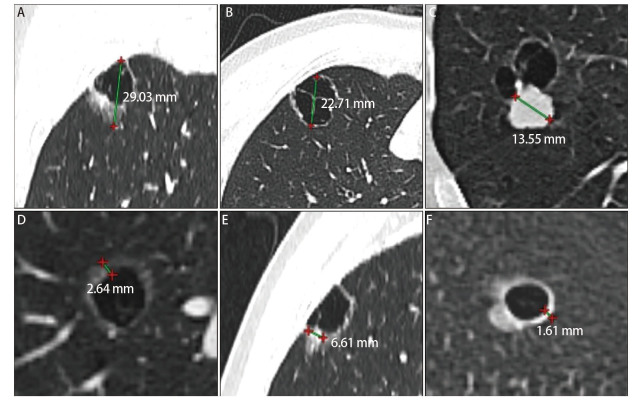
囊腔型肺腺癌影像学测量标准。A：病灶直径测量为肺窗上病灶在MPR的最大轴向截面的最大直径；B：囊腔直径测量为囊腔在MPR的最大轴向截面的最大直径；C：团块状结节直径测量为邻接囊腔的结节的最大直径；D：囊壁环形型结节直径测量为从囊腔边界到GGO或实变边界的最大距离，垂直于囊腔切线；E: 团块状实性成分直径测量为实性成分在MPR的最大轴向截面的最大直径；F：环壁状实性成分直径测量为垂直于囊腔切线的最大实性壁厚。

综合以上影像学特征与指标，我们参照上海市肺科医院提出的囊腔型肺癌形态学分类系统^[1]^，将本次研究对象划分为四类病灶（图3）：I型表示囊腔大小<6.5 mm的纯磨玻璃结节；II型表示囊腔大小≥6.5 mm的纯磨玻璃结节；III型表示囊腔伴部分实性结节；IV型表示囊腔伴纯实性结节。

**图 3:**
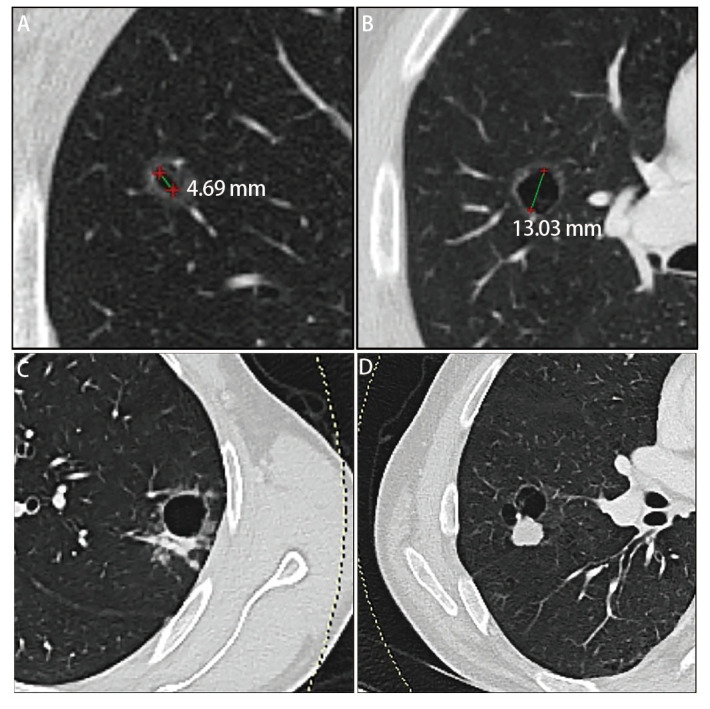
CT图像分类的图例。A：I型表示囊腔大小<6.5 mm的纯磨玻璃结节；B：II型表示囊腔大小≥6.5 mm的纯磨玻璃结节；C：III型表示囊腔伴部分实性结节；D：IV型表示囊腔伴纯实性结节。

### 1.5 其余临床资料的获取

除上述影像资料外，临床多特征资料还包括基线信息以及肿瘤标志物。其中，基线信息包括年龄、性别、吸烟史、良性肺病史、肺气肿史和肺外肿瘤史。肿瘤标志物包含甲胎蛋白（alpha-fetoprotein, AFP）、癌胚抗原（carcinoembryonic antigen, CEA）、糖类抗原199（carbohydrate antigen 199, CA199）、糖类抗原724（carbohydrate antigen 724, CA724）、细胞角蛋白19片段（cytokeratin 19 fragment antigen 21-1, CYFR21-1）与神经元特异性烯醇化酶（neuron-specific enolase, NSE）。

### 1.6 统计分析

本研究对于基线资料、影像学资料与肿瘤标志物资料等临床多特征资料进行单因素分析、LASSO回归与多因素Logistic回归分析。数据分析软件使用SPSS 26.0与R 4.2.1。在单因素分析中，计量资料首先用K-S法检验正态性，正态/非正态分布分别采取独立样本t检验/Mann-Whitney U检验进行比较，以均数±标准差或中位数[M（P25, P75）]描述；计数资料组间比较采用卡方检验或Fisher精确检验，以频数或百分比（%）进行描述。对于单因素分析中差异有统计学意义的因素采用LASSO回归进行降维处理，筛选出具有代表性的风险因素。其中计量资料通过受试者工作特征（receiver operating characteristic, ROC）曲线分析，根据Youden指数最大值选取最佳cut-off值，将其转换为计数资料，然后纳入多因素Logistic回归分析，探究囊腔型肺腺癌浸润性的独立危险因素，建立浸润性风险预测模型，绘制ROC曲线，计算曲线下面积（area under the curve, AUC），比较各独立危险因素与本预测模型的预测性能，P<0.05为差异具有统计学意义。

## 2 结果

### 2.1 囊腔型肺腺癌浸润前组与浸润组病理、病灶分型及手术方式比较

浸润前组共计47例，其中，AAH 2例（4.26%），AIS 8例（17.02%），MIA 37例（78.72%）。浸润组共82例，均为IAC（100.00%）。两组的病理分类、病灶分型与手术方式均存在统计学差异（P<0.05），见表1。

**表 1:** 囊腔型肺腺癌浸润前组与浸润组病理、病灶分型及手术方式比较

Features	Pre-invasive (n=47)	Invasive (n=82)	P
Pathology			<0.001
AAH	2 (4.26%)	0 (0.00%) 0 (0.00%) 0 (0.00%)	
AIS	8 (17.02%)		
MIA	37 (78.72%)		
IAC	0 (0.00%)	82 (100.0%)	
Classification			<0.001
Type I	18 (38.30%)	8 (9.76%)	
Type II	12 (25.53%)	14 (17.07%)	
Type III	16 (34.04%)	32 (39.02%)	
Type IV	1 (2.13%)	28 (34.15%)	
Surgical approach			<0.001
Wedge resection	14 (29.79%)	5 (6.10%)	
Segmentectomy	22 (46.81%)	20 (24.39%)	
Lobectomy	11 (23.40%)	57 (69.51%)	

### 2.2 囊腔型肺腺癌浸润性单因素分析

对比分析囊腔型肺腺癌浸润前组与浸润组的各项纳入因素，其中性别、年龄、肺气肿史、吸烟史、NSE、囊腔数、结节直径、病灶直径、实性成分直径、囊腔直径、囊壁结节、短毛刺征、囊壁光滑程度、分叶征、胸膜牵拉、囊腔形状、血管穿行与支气管穿行均有统计学意义（P<0.05），见表2。

**表 2:** 囊腔型肺腺癌浸润前组与浸润组单因素分析

Features		Pre-invasive (n=47)	Invasive (n=82)	P
Age (yr)		51.23±14.96	61.27±11.74	<0.001
Gender	Female	28 (59.57%)	22 (26.83%)	<0.001
	Male	19 (40.43%)	60 (73.17%)	
Smoking history	None	41 (87.23%)	48 (58.54%)	<0.001
	Yes	6 (12.77%)	34 (41.46%)	
History of lung disease	None	46 (97.87%)	79 (96.34%)	>0.999
	Yes	1 (2.13%)	3 (3.66%)	
History of emphysema	None	44 (93.62%)	64 (78.05%)	0.025
	Yes	3(6.38%)	18 (21.95%)	
History of extrapulmonary tumors	None	44 (93.62%)	79 (96.34%)	0.668
	Yes	3 (6.38%)	3 (3.66%)	
Tumour location	LUL	8 (17.02%)	20 (24.39%)	0.529
	LLL	6 (12.77%)	13 (15.85%)	
	RUL	14 (29.79%)	27 (32.93%)	
	RML	4 (8.51%)	3 (3.66%)	
	RLL	15 (31.91%)	19 (23.17%)	
Depth ratio		24.11%±12.97%	25.01%±12.69%	0.700
Region	Middle	10 (21.28%)	24 (29.27%)	0.407
	Outer	37 (78.72%)	58 (70.73%)	
Number of cystic airspaces	Single	37 (78.72%)	30 (36.59%)	<0.001
	Multiple	10 (21.28%)	52 (63.41%)	
Lesion diameter (mm) [M (P25, P75) ]		12.30 (10.20, 15.90)	22.00 (17.10,27.05)	<0.001
Cystic cavity diameter (mm) [M (P25, P75) ]		4.63 (3.61, 7.22)	7.11 (5.29, 11.15)	<0.001
Nodule diameter (mm) [M (P25, P75) ]		4.68 (3.51, 6.51)	8.29 (6.06, 13.13)	<0.001
Solid components diameter (mm) [M (P25, P75) ]		0.00 (0.00, 1.66)	4.18 (0.00, 9.05)	<0.001
Cyst wall nodule	None	35 (74.47%)	21 (25.61%)	<0.001
	Yes	12 (25.53%)	61 (74.39%)	
Smoothness of cyst wall	Smooth	35 (74.47%)	22 (26.83%)	<0.001
	Rough	12 (25.53%)	60 (73.17%)	
Shape of cystic airspace	Oval	36 (76.60%)	33 (40.24%)	<0.001
	Irregular	11 (23.40%)	49 (59.76%)	
Lobulation	None	37 (78.72%)	22 (26.83%)	<0.001
	Yes	10 (21.28%)	60 (73.17%)	
Short burr sign	None	21 (44.68%)	13 (15.85%)	<0.001
	Yes	26 (55.32%)	69 (84.15%)	
Plueral retraction	None	37 (78.72%)	29 (35.37%)	<0.001
	Yes	10 (21.28%)	53 (64.63%)	
Vascular penetration	None	10 (21.28%)	5 (6.10%)	0.012
	Yes	37 (78.72%)	77 (93.90%)	
Bronchial penetration	None	42 (89.36%)	52 (63.41%)	0.002
	Yes	5 (10.64%)	30 (36.59%)	
AFP	Normal	47 (100.00%)	81 (98.78%)	>0.999
	Abnormal	0 (0.00%)	1 (1.22%)	
CEA	Normal	42 (89.36%)	64 (78.05%)	0.151
	Abnormal	5 (10.64%)	18 (21.95%)	
CA199	Normal	47 (100.00%)	77 (93.90%)	0.158
	Abnormal	0 (0.00%)	5 (6.10%)	
CA724	Normal	45 (95.74%)	76 (92.68%)	0.710
	Abnormal	2 (4.26%)	6 (7.32%)	
CYFR21-1	Normal	44 (93.62%)	74 (90.24%)	0.745
	Abnormal	3 (6.38%)	8 (9.76%)	
NSE	Normal	31 (65.96%)	37 (45.12%)	0.028
	Abnormal	16 (34.04%)	45 (54.88%)	

LUL: left upper lung; LLL: left lower lung; RUL: right upper lung; RML: right middle lung; RLL: right lower lung; AFP: alpha-fetoprotein; CEA: carcinoembryonic antigen; CA199: carbohydrate antigen 199; CA724: carbohydrate antigen 724; CYFR21-1: cytokeratin 19 fragment antigen 21-1; NSE: neuron-specific enolase.

### 2.3 LASSO回归筛选处理

通过LASSO回归将有统计学意义的单因素分析变量降维处理，经10折交叉验证最小均方误差的λ=0.028，最终对应模型筛选出10个变量：性别、年龄、吸烟史、NSE、囊腔数、病灶直径、囊腔直径、囊壁结节、囊壁光滑程度与分叶征。

### 2.4 囊腔型肺腺癌浸润性多因素Logistic回归分析

将LASSO回归筛选的变量纳入多因素Logistic回归分析中，以α=0.05为独立危险因素检验标准。其中，将年龄、病灶直径与囊腔直径计量资料选取Youden指数最大处的cut-off值进行计数资料转换，各计量资料因素的cut-off值与分类如下：年龄（55.5岁，≤55.5岁/>55.5岁）、病灶直径（17.85 mm，≤17.85 mm/>17.85 mm）、囊腔直径（5.17 mm，≤5.17 mm/>5.17 mm）。研究发现有囊壁结节（P=0.035）与有分叶征（P=0.001）是囊腔型肺腺癌浸润性的独立危险因素（表3）。

**表 3:** 囊腔型肺腺癌浸润性的多因素Logistic回归分析

Features	RC	OR	95%CI	P
Age (>55.5 yr)	0.026	1.027	0.983-1.073	0.240
Gender (Male)	0.372	1.451	0.350-6.020	0.608
Smoking history (Yes)	1.268	3.552	0.668-18.890	0.137
Number of cystic airspaces (Multiple)	0.571	1.770	0.386-8.111	0.462
NSE (Abnormal)	0.799	2.224	0.637-7.766	0.210
Lesion diameter (>17.85 mm)	0.160	1.173	0.998-1.380	0.054
Cystic cavity diameter (>5.17 mm)	0.168	1.183	0.053-2.015	0.186
Cyst wall nodule (Yes)	1.476	4.376	1.111-17.231	0.035
Smoothness of cyst wall (Rough)	0.453	1.573	0.017-6.723	0.577
Lobulation (Yes)	2.407	11.102	2.841-43.382	0.001

RC: regression coefficient; OR: odds ratio; CI: confidence interval.

### 2.5 ROC曲线与预测模型的建立

根据多因素分析结果（表3），我们选取对应因素的回归系数（regression coefficient, RC），绘制ROC曲线（图4）并建立预测模型：P=e^x/（1+e^x），x=-7.927+1.476*囊壁结节+2.407*分叶征。其中P为浸润性预测值概率，e为自然对数，因素结果有则记为1，无则记为0：有囊壁结节记为1，无囊壁结节记为0；有分叶征记为1，无分叶征记为0。各变量对于ROC曲线的AUC均大于0.5。囊壁结节的AUC=0.744（95%CI: 0.654-0.835），标准误（standard error, SE）为0.046；分叶征的AUC=0.759（95%CI: 0.672-0.847），SE为0.045；本预测模型的AUC=0.950（95%CI: 0.915-0.984），SE为0.018，较上述两变量预测效能佳。

**图 4:**
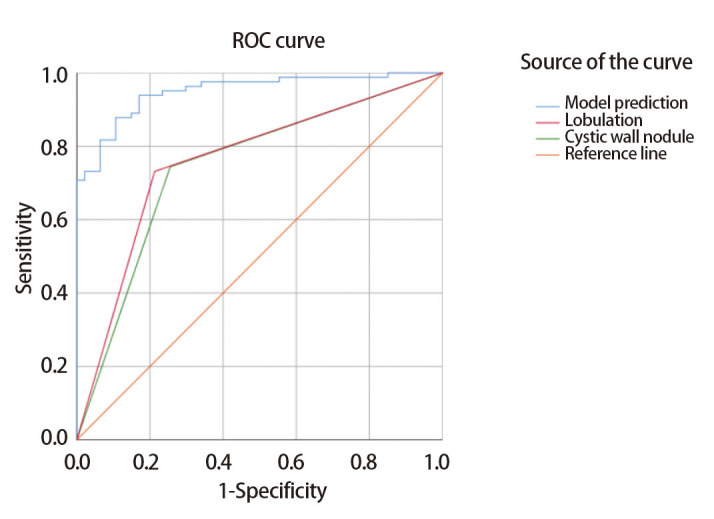
基于多因素回归结果绘制的ROC曲线

## 3 讨论

囊腔型肺癌是一类特殊的肺癌，其中囊腔型肺腺癌最为常见^[9]^。肺肿瘤的浸润性是患者能否长期生存的重要影响因素。不同浸润程度的预后存在巨大差异^[10]^，且浸润性高的肺癌病灶往往选择更大切除范围^[15]^，这与本次回顾性研究结果一致：对于浸润前组我中心多采用楔形切除和肺段切除，而对于浸润组多采用肺段切除和肺叶切除，这反映我中心对囊腔型肺腺癌浸润性的认识程度，对于浸润性高的病灶需切除更大的范围。因此术前识别并评估囊腔型肺腺癌的浸润性至关重要。传统评估模型如VA模型^[16]^、Brock模型^[17]^等，均具有一定局限性，无法准确评估。于是我们综合了Zhu等^[1]^、Farooqi等^[5]^、Mascalchi等^[12]^多位学者的研究成果，结合多项临床特征，建立了一个新型预测模型，在浸润性预测上展示出了高效能（AUC=0.950）。

通过分析，我们发现囊壁结节与分叶征是囊腔型肺腺癌浸润性的独立危险因素。囊壁结节是指囊壁上形成的结节，其影像学可表现为实性成分或磨玻璃成分。Farooqi等^[5]^的研究对26例恶性囊腔型肺癌患者进行观察，发现初期的单纯薄壁病灶后期均出现囊壁结节；Shen等^[18]^亦探究发现囊腔型肺癌若伴有囊壁结节，可能肿瘤浸润性更高且预后生存率更低。这说明了囊壁结节有可能是囊腔型恶性病灶的一个阶段表现。分叶征指的是肿瘤非囊腔成分表面呈波浪状或扇形结构的特征。多篇文献^[1,13,18,19]^报道了分叶征对于囊腔型肺腺癌的预测价值，它可能与肿瘤组织向各方向生长速度不一致或者生长过程中某一方向受阻有关。Jung的研究^[20]^随访了27例囊腔型肺癌患者的CT影像资料，总结出囊腔型肺癌发展的4个阶段：（1）实性结节中出现囊腔；（2）囊腔增大；（3）囊腔边缘出现实性成分；（4）实性成分逐步包绕囊腔并且增厚。这体现出其浸润性增加的过程。对比影像学形态改变，我们发现：囊壁结节可能出自上述（3）阶段，分叶征则可能出现在上述（4）阶段。当囊腔型肺腺癌开始出现囊壁结节，提示了肿瘤组织进入了生长阶段；分叶征则是肿瘤组织进一步生长且各方向生长速度不一致的标志，其生物学行为表现为肿瘤组织进入浸润性生长阶段，浸润性相对更高。这与我们的研究相互印证，在统计上表现为分叶征的比值比（odds ratio, OR）值（OR=11.102）大于囊壁结节（OR=4.376），说明分叶征较囊壁结节对囊腔型肺腺癌浸润性预测的风险性更高。它们的演变发展可为囊腔型肺腺癌的纵向观测提供一定参考依据。

除上述两个独立危险因素，本研究部分因素在单因素分析中也呈现显著差异。囊腔型肺癌男性患病率远高于女性^[21,22]^，这可能与吸烟、肺气肿、良性肺病等相关危险因素在男性群体中的高发生率有关。Goldstein等^[23]^、Araki等^[24]^学者针对这些因素分别提出支气管顺应性变化假说、致癌物质沉积假说等。这些高危因素使得男性与吸烟史两个因素在单因素分析中较为显著。据报道^[5,7,25,26]^，囊腔型肺腺癌可发生于任何肺叶，且多位于肺外周，这可能与此类病灶多累及外周小气道，肿瘤组织生长侵及小气道后形成气体流通的单向通道，继而形成囊腔。本研究对囊腔型肺腺癌病灶的分带与相对深度进行数值测量处理，发现尚有一部分病灶位于肺野中带相对较深位置，尚不能排除其原发于中带的可能，之前囊腔型肺癌外周小气道“单向阀”形成假说需要进一步的验证。

囊腔型肺腺癌的影像特征多样，与普通肺结节存在异同。结节大小、实性成分大小已可用于常规肺结节的浸润性预测^[27,28]^，然而囊腔型肺癌病灶同时存在囊腔与结节，二者关系复杂多变。常规肺癌肿瘤原发灶-淋巴结-转移（tumor-node-metastasis, TNM）分期中的T分期测量不可避免地受到本类病灶囊腔的影响，不能准确提示肿瘤组织大小。本次研究对于病灶直径、结节直径、囊腔直径、实性成分直径分别进行系统测量及数据分析，此四种因素在囊腔型肺腺癌浸润前组与浸润组单因素分析中存在显著差异。上海市胸科医院的研究得出类似的结论，且测量数据均值和分布与本研究相近^[1]^。此外，Detterbeck等^[21]^建议，在实性成分出现或进展时需要进行干预，其研究中所有伴随囊腔的纯实性结节均为浸润性病变。因此实性成分直径对于囊腔型腺癌的浸润性具有重要预测价值。毛刺征是肿瘤细胞向周围组织浸润生长并牵拉周围的组织而表现出的一种恶性特征，高度提示结节恶性可能，其中短毛刺征在肺腺癌中发生率为81%-100%^[29]^。本研究亦得到相似比例，对于囊腔型肺腺癌具有一定预测价值。在Tan等^[30]^研究囊腔型肺癌病理时发现，在恶性囊腔型肺病灶中，具有分隔的多囊腔病灶占到58.4%。Wang等^[31]^也报道，基于囊腔数量的分类可预测良恶性。CT上囊腔的分隔对应于纤维组织或穿过的气道或血管，囊腔数量与血管穿行、支气管穿行在影像学上表现息息相关，这些因素均为单因素分析下有统计学意义的因素，但在多因素分析中不显著，这可能因为相关因素彼此间存在间接关联，影响多因素分析结果。囊腔数量、血管穿行与支气管穿行对囊腔型肺腺癌浸润性的预测价值需要证实。

本研究还纳入了肿瘤标志物资料进行探讨。有研究^[32]^表明，CYFRA21-1和NSE在肺癌诊断中显示出较高的敏感性和特异性，但在本研究中仅有NSE是单因素分析下的危险因素，相关肿瘤标志物与囊腔型肺腺癌的关系有待进一步研究。近年来，免疫组化、基因突变等非影像学资料开始被逐步纳入到肺癌的综合性评估中，关于影像学的前沿研究也在不断进展。目前，已有多篇文章对正电子发射计算机断层显像（positron emission tomography/CT, PET/CT）的检测效能以及其示踪剂氟代脱氧葡萄糖（fluorodeoxyglucose, FDG）的摄取展开讨论，结论不一^[33,34]^，其诊断效能有待进一步检验。未来，相关领域发展空间巨大。

本研究存在的局限性及后期改进措施：本研究以回顾性形式开展，存在一定的病例选择偏倚，囊腔型肺癌的发病率较低以及仅在单中心收集研究数据导致样本量有限，其预测模型缺乏验证。后续我们将扩大样本量并进行预测模型的验证，同时纳入预后数据，进一步评估囊腔型肺腺癌患者的预后生存情况。

综上所述，通过分析囊腔型肺腺癌的临床多特征，发现了囊壁结节与分叶征为囊腔型肺腺癌浸润性的独立危险因素，建立浸润性风险预测模型：P=e^x/（1+e^x），x=-7.927+1.476*囊壁结节+2.407*分叶征，AUC=0.950，可为临床诊治工作提供参考与帮助。
